# In Situ Non-Destructive Stiffness Assessment of Fiber Reinforced Composite Plates Using Ultrasonic Guided Waves

**DOI:** 10.3390/s24092747

**Published:** 2024-04-25

**Authors:** Maarten Adams, Arnaud Huijer, Christos Kassapoglou, Johannes A. A. Vaders, Lotfollah Pahlavan

**Affiliations:** 1 Department of Maritime and Transport Technology, Delft University of Technology, Mekelweg 2, 2628 CD Delft, The Netherlands; a.j.huijer@tudelft.nl (A.H.); l.pahlavan@tudelft.nl (L.P.); 2 Department of Aerospace Structures and Materials, Delft University of Technology, Kluyverweg 1, 2629 HS Delft, The Netherlands; c.kassapoglou@tudelft.nl; 3 Materiel and IT Command, The Netherlands Ministry of Defence, Kromhout Kazerne, Herculeslaan 1, 3584 AB Utrecht, The Netherlands; jaa.vaders@mindef.nl

**Keywords:** ultrasonic guided waves, structural stiffness assessment, glass fiber-reinforced composites, semi-analytical finite element method

## Abstract

The multimodal and dispersive character of ultrasonic guided waves (UGW) offers the potential for non-destructive evaluation of fiber-reinforced composite (FRC) materials. In this study, a methodology for in situ stiffness assessment of FRCs using UGWs is introduced. The proposed methodology involves a comparison between measured wave speeds of the fundamental symmetric and antisymmetric guided wave modes with a pre-established dataset of UGW speeds and translation of them to corresponding stiffness properties, i.e., ABD-components, in an inverse manner. The dispersion relations of guided waves have been calculated using the semi-analytical finite element method. First, the performance of the proposed methodology has been assessed numerically. It has been demonstrated that each of the independent ABD-components of the considered laminate can be approximated with an error lower than 10.4% compared to its actual value. The extensional and bending stiffness properties can be approximated within an average error of 3.6% and 9.0%, respectively. Secondly, the performance of the proposed methodology has been assessed experimentally. This experimental assessment has been performed on a glass fiber-reinforced composite plate and the results were compared to mechanical tensile and four-point bending tests on coupons cut from the plate. Larger differences between the estimated ABD-components according to UGW and mechanical testing were observed. These differences were partly attributed to the variation in material properties across the test plate and the averaging of properties over the measurement area.

## 1. Introduction

Fiber-reinforced composite (FRC) materials have been gaining great popularity in marine structures over the past couple of decades because of their excellent strength-to-weight ratio [1,2], low density [3], corrosion resistance [1], and additional degrees of freedom in the design process [4]. However, the non-isotropic material properties in combination with rather complex manufacturing procedures provoke uncertainties in material properties and structural integrity of FRC materials after production and during their use [5,6]. Process-induced defects, such as voids, fiber misalignment, and delamination are common problems encountered during composite manufacturing [7,8,9]. The formation of these irregularities can significantly affect the mechanical performance of FRCs [9,10,11,12]. Moreover, structural degradation arises during service of the structure due to (cyclic) loading, the operating environment, and/or human errors.

To fully exploit the advantages of FRC materials, non-destructive evaluation (NDE) techniques have been proposed to analyze structural properties and identify damage [13]. These techniques utilize mechanical, chemical, or electromagnetic forces to disrupt the structure and measure the response. By anticipating that any internal irregularity will alter the returned signal, this signal offers insight into the material properties or structural damage [14]. Commonly used techniques for damage detection and structural integrity assessment are visual inspection and tap testing [15], radiographic testing [16,17], electromagnetic testing [18,19,20], shearography [21], vibration-based testing [22], acoustic emission testing [23,24], and ultrasonic testing [25,26,27,28,29,30]. However, these inspection methods are often limited by their insensitivity to small forms of damage and/or by their inability to detect damage that is not in close proximity to the inspection point. Additionally, these methods may not accurately assess the severity of the detected damage, making it difficult to determine the appropriate course of action for repairs or mitigation [31].

Over the past decade, ultrasonic guided wave (UGW) inspection methods have emerged as a promising technique for NDE due to their notable advantages. These methods offer a cost-effective, rapid, and repeatable means of inspecting large areas in a short amount of time without requiring the motion of transducers. UGWs are sensitive to small-size damage and can quantitatively evaluate both surface and internal damages that have a size greater than half its wavelength. Additionally, UGW devices can have a low power consumption, making them well-suited for use in remote or hard-to-reach locations [31,32,33].

The multimodal and dispersive character of UGW propagation is sensitive to the structural properties and has therefore been the basis of multiple studies on damage detection [29,32,34,35,36,37] and elastic properties characterization [38,39,40] of FRCs. Combining these features with their non-destructive nature shows the high potential of UGWs in the field of NDE [41,42]. Several studies have been conducted on the stiffness determination of FRC materials using UGWs [39,43]. Most of these methods involve a computationally intensive optimization process between the results obtained from experiments and the predictions generated by a forward numerical model [44].

This study introduces an enhanced non-destructive method for in situ stiffness assessment of FRCs using UGWs. The proposed methodology utilizes a computationally efficient inversion algorithm to evaluate the structural stiffness of FRCs by comparing experimentally measured UGW speeds with a pre-established dataset of UGW speeds and corresponding stiffness properties. The approach offers potential for future rapid in situ assessment of large-scale composite structures.

The performance of the proposed methodology was initially evaluated through a numerical evaluation. To demonstrate its practical feasibility for in situ applications, a glass fiber reinforced sample plate was fabricated and subjected to the assessment methodology. Subsequently, the plate was cut into coupons and mechanical tests were conducted to evaluate the stiffness properties obtained from this new assessment methodology.

The proposed methodology and evaluation procedure are described in Section 2. The experiments consisting of UGW testing and mechanical testing are discussed in Section 3. The results and discussion are presented in Section 4. Lastly, Section 5 presents the conclusions.

## 2. Methodology

The zero-order symmetric ($S_{0}$) and antisymmetric ($A_{0}$) wave modes are most often used in guided wave NDE techniques [45,46]. The main reason for that is their sensitivity to structural damage and strong correlation to mechanical stiffness [35,36,47]. Next to that, these wave modes are more straightforward to excite and measure than the higher-order guided wave modes.

The classical laminate theory (CLT) is commonly used to describe the behavior of composite materials under different types of loading conditions through use of the ABD-matrix, as described in Equation (1) [4].

(1) $$\begin{array}{c} \left\{\begin{array}{c} \left\{N\right\}\\ \left\{M\right\} \end{array}\right\}=\left[\begin{array}{cc} \left[A\right] & \left[B\right]\\ \left[B\right] & \left[D\right] \end{array}\right]\left\{\begin{array}{c} \left\{\epsilon^{0}\right\}\\ \left\{\kappa^{0}\right\} \end{array}\right\} \end{array}$$

Here, {N} and {M} are the external forces and moments applied on the structure, respectively, $\left\{\epsilon^{0}\right\}$ and $\left\{\kappa^{0}\right\}$ denote the internal strains and curvatures. $A_{ij}$ represents the in-plane stiffnesses, $B_{ij}$ captures the coupling between in-plane forces and out-of-plane deformations, and $D_{ij}$ signifies the out-of-plane bending stiffnesses. Using these ABD stiffness components, a generic expression for group wave propagation in anisotropic media is formulated as:

(2) $$\begin{array}{c} c_{g}=G\left(m,\omega ,A_{ij},B_{ij},D_{ij},I_{0},I_{1},I_{2}\right) \end{array}$$

Here, *m* denotes the guided wave mode, ω denotes the wave frequency, and $I_{0}$, $I_{1}$, and $I_{2}$ denote the first, second, and third mass moments of inertia, describing the total mass, center of mass, and moment of inertia, respectively. Generally, it can be expected that symmetric wave modes are predominantly influenced by the extensional stiffness, while antisymmetric wave modes are dominated by bending stiffness.

Establishing an analytical solution for Equation (2) is not deemed feasible due to the complexity of the governing equations for guided waves in anisotropic media. This research investigates the possibility of utilizing an approximate description of $c_{g}$ in terms of the ABD-components using a set of coupling coefficients ($c_{i}$). For an arbitrary guided wave mode *m* propagating at frequency ω, this relationship is given as

(3) $$\begin{array}{c} c_{m,1}A_{11}+c_{m,2}A_{12}+c_{m,3}A_{16}+\cdots +c_{m,S}D_{66}=c_{g,m}^{2}+e_{m} \end{array}$$

Here, subscript *S* denotes the total number of unknown ABD-components and $e_{m}$ denotes the approximation error. This error is dependent on the wave mode, material properties, and wave frequency, and may not be considered generally negligible. When dealing with symmetric wave modes, the error is expected to be fairly small for laminates with weak axial-bending coupling. However, for antisymmetric wave modes, a larger error may be expected, as the relationship between material stiffness and $c_{g}$ is generally more complex and involves higher-order terms [48,49]. Increased axial-bending coupling is expected to further increase the approximation error. Equation (3) can also be expressed in matrix-vector format:

(4) $$\begin{array}{c} \left[C\right]{\left\{\Psi \right\}}^{T}=\left\{c_{g}^{2}\right\}+\left\{e\right\} \end{array}$$

In this system of equations, $\left[C\right]$ represents the matrix of coupling coefficients, while $\left\{c_{g}^{2}\right\}$ and {e} denote the vectors containing the squared group speeds and approximation errors, respectively. At sufficiently low frequencies where only the $S_{0}$ and $A_{0}$ wave modes are involved, the vector $\left\{c_{g}^{2}\right\}$ reduces to

(5) $$\begin{array}{c} \left\{c_{g}^{2}\right\}={\left\{\begin{array}{ccccccccccc} c_{g,S_{0},1}^{2} & c_{g,S_{0},2}^{2} & c_{g,S_{0},j}^{2} & \cdots & c_{g,S_{0},W}^{2} & | & c_{g,A_{0},1}^{2} & c_{g,A_{0},2}^{2} & c_{g,A_{0},j}^{2} & \cdots & c_{g,A_{0},W}^{2} \end{array}\right\}}^{T} \end{array}$$

Here, subscript *W* indicates the total number of $S_{0}$ and $A_{0}$ wave velocities included. Vector {Ψ} (size $\left[1\times S\right]$) in Equation (4) represents the unknown stiffness properties of the FRC plate under analysis, structured as

(6) $$\begin{array}{c} \left\{\Psi \right\}=\left\{\begin{array}{cccccccccccccc} A_{11} & A_{12} & \cdots & A_{66} & | & B_{11} & B_{12} & \cdots & B_{66} & | & D_{11} & D_{12} & \cdots & D_{66} \end{array}\right\} \end{array}$$

Based on this system, it would be possible to estimate {Ψ} using an inverse procedure when matrix $\left[C\right]$ (size [2W×S]) is known and the squared group speed vector $\left\{c_{g}^{2}\right\}$ (size $\left[2W\times 1\right]$) is obtained through measurements.

### 2.1. Calculation of the Coupling Coefficients

To calculate the coupling coefficients ($c_{m,i}$) in matrix $\left[C\right]$, a specific composite plate of interest is considered. The design process for composite laminates allows for a wide range of possible stiffness properties resulting from design properties, such as material type, stacking sequence, and plate/ply thickness. By utilizing prior information (for example, a known stacking sequence and/or $E_{1}$ ply stiffness) of the plate of interest, this wide range of possible stiffness properties can be narrowed down to a reduced range of stiffness possibilities. The proposed method captures this range of stiffness possibilities in the coupling coefficients. To achieve this, the coefficients are numerically determined by analyzing a set of *R* reference laminates $p_{r}$, where 1≤r≤R. These reference laminates are chosen so that their stiffness properties fall within the range of stiffness possibilities. By using a sufficient number of reference laminates to sufficiently cover the range of stiffness possibilities, it is expected that a converged stiffness approximation can be obtained. Determination of the set of coupling coefficients $\left\{c_{m}\right\}$ related to wave mode *m* (Equation (3)) is described as follows:

(7) $$\begin{array}{c} \Psi_{\mathrm{ref}}\left\{c_{m}\right\}=\left\{c_{g,m,\mathrm{ref}}^{2}\right\} \end{array}$$

where

(8) $$\begin{array}{c} \Psi_{\mathrm{ref}}=\left[\begin{array}{ccccc} A_{11,p_{1}} & A_{12,p_{1}} & A_{16,p_{1}} & \cdots & D_{66,p_{1}}\\ A_{11,p_{2}} & A_{12,p_{2}} & A_{16,p_{2}} & \cdots & D_{66,p_{2}}\\ A_{11,p_{r}} & A_{12,p_{r}} & A_{16,p_{r}} & \cdots & D_{66,p_{r}}\\ \vdots & \vdots & \vdots & & \vdots \\ A_{11,p_{R}} & A_{12,p_{R}} & A_{16,p_{R}} & \cdots & D_{66,p_{R}} \end{array}\right] \end{array}$$

(9) $$\begin{array}{c} \left\{c_{m}\right\}=\left\{\begin{array}{ccc} c_{m,1} & c_{m,2} & \cdots c_{m,S} \end{array}\right\}z^{T} \end{array}$$

(10) $$\begin{array}{c} \left\{c_{g,m,\mathrm{ref}}^{2}\right\}={\left\{\begin{array}{ccccc} c_{g,m.p_{1}}^{2} & c_{g,m,p_{2}}^{2} & c_{g,m,p_{r}}^{2} & \cdots & c_{g,m,p_{R}}^{2} \end{array}\right\}}^{T} \end{array}$$

Here, each row of matrix $\Psi_{\mathrm{ref}}$ (size $\left[R\times S\right]$) consists of the ABD-components of a single reference laminate $p_{r}$, which is calculated using the CLT. Similarly, each element of vector $\left\{c_{g,m,\mathrm{ref}}^{2}\right\}$ (size $\left[R\times 1\right]$) consists of the squared group speed of wave mode *m* of reference laminate $p_{r}$. Equation (7) is solved in a least-squares sense.

There is generally a large difference in magnitude of the extensional stiffness components $A_{ij}$, coupling stiffness components $B_{ij}$, and bending stiffness components $D_{ij}$. To improve the condition of the numerical operations, matrix $\Psi_{\mathrm{ref}}$ is column-wise normalized by the absolute maximum component included in the column. This matrix scaling can be expressed as

(11) $$\begin{array}{c} {\bar{\Psi }}_{\mathrm{ref}}=\Psi_{\mathrm{ref}}⊙\left(1/\Psi_{\mathrm{ref},\max}\right) \end{array}$$

Here, vector $\Psi_{\mathrm{ref},\max}$ contains the absolute maximum stiffness component of each column of matrix $\Psi_{\mathrm{ref}}$ and ⊙ indicates the element-wise matrix multiplication. This results in the following modified version of Equation (7):

(12) $$\begin{array}{c} {\bar{\Psi }}_{\mathrm{ref}}\left\{c_{m}\right\}=\left\{c_{g,m,\mathrm{ref}}^{2}\right\} \end{array}$$

Consequently, vector {Ψ} in Equation (4) is column-wise normalized, resulting in the following modification of Equation (4):

(13) $$\begin{array}{c} \left[C\right]{\left\{\bar{\Psi }\right\}}^{T}=\left\{c_{g}^{2}\right\} \end{array}$$

where

(14) $$\begin{array}{c} \left\{\bar{\Psi }\right\}=\left\{\Psi \right\}⊙1/\left(\Psi_{\mathrm{ref},\max}\right) \end{array}$$

#### Dispersion Analysis Using the Semi-Analytical Finite Element Method

**Figure 1:**
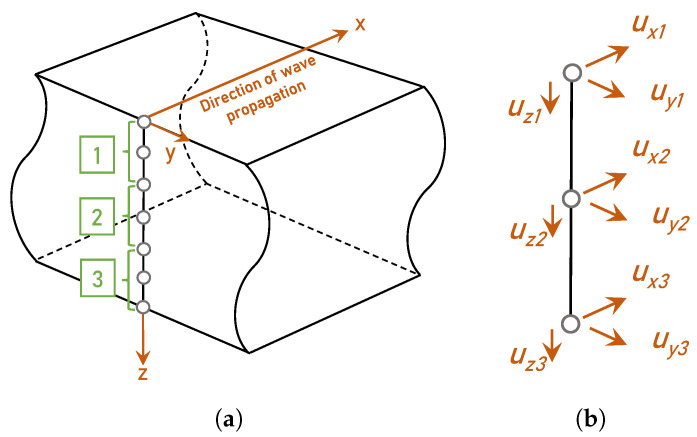
(**a**) Schematic representation of SAFEM for wave propagation in *x*-direction. (**b**) Degrees of freedom of the *i*th node element.

The reference velocities in $\left\{c_{g,m,\mathrm{ref}}^{2}\right\}$ (Equation (12)) are calculated from $\Psi_{\mathrm{ref}}$ by using the semi-analytical finite element method (SAFEM). SAFEM is a particularly efficient tool for calculating phase and group speed dispersion curves of guided waves in multilayered composite laminates and is commonly used as forward numerical model in NDE [50,51,52,53,54,55]. SAFEM operates under the assumption of plane strain behavior, employing finite element discretization along the thickness direction or cross section of the waveguide. The displacement in the direction of wave propagation is analytically described using harmonic exponential functions. This makes it more computationally efficient than conventional 3D FEM [56]. Figure 1 shows a discretization of wave propagation in the *x*-direction used in 1D SAFEM, assuming an infinitely wide plate and three-node elements. The equations of motion are expressed by Hamilton’s equation [57] and the SAFEM solutions are obtained in a stable manner from an eigenvalue problem. A detailed description of SAFEM is provided by Barazanchy [50] and Bartoli [51].

### 2.2. Robustness of the Algorithm

When applied in practice, the measured squared group speed vector $\left\{c_{g}^{2}\right\}$ (used in Equation (13)) may be affected by environmental conditions and/or measurement errors. To study the robustness of the algorithm with respect to imperfect input data, a numerical sensitivity study is performed. In this sensitivity study, different system configurations are considered. These configurations vary in the number of unknown ABD-components (*S*) included in Equation (13) and are discussed in Section 2.3.3. For each configuration the effect of the presence of measurement errors on the approximation of {Ψ} is studied. The group speed vector, including measurement errors $\left\{c_{g,\mathrm{ME}}\right\}$, is defined as follows:

(15) $$\begin{array}{c} \left\{c_{g,\mathrm{ME}}\right\}=\left\{c_{g}\right\}+\left\{\Delta c_{g}\right\} \end{array}$$

Here, vector $\left\{c_{g}\right\}$ is the original group speed vector as defined in Equation (13). Vector $\left\{\Delta c_{g}\right\}$ includes the measurement errors and is calculated as:

(16) $$\begin{array}{c} \left\{\Delta c_{g}\right\}=\left\{c_{g}\right\}⊙\left\{f_{\mathrm{ME}}\right\} \end{array}$$

Here, the original velocity vector is element-wise multiplied by error vector $\left\{f_{\mathrm{ME}}\right\}$ defined as:

(17) $$\begin{array}{c} \left\{f_{\mathrm{ME}}\right\}={\left\{\begin{array}{ccccc} e_{1} & e_{2} & e_{i} & \cdots & e_{2W} \end{array}\right\}}^{T}\;\;\;\;\mathrm{where},\;\;-E_{max}\leq e_{i}\leq E_{max} \end{array}$$

Here, $e_{i}$ denotes an arbitrary value between $-E_{max}$ and $+E_{max}$, which defines the maximum possible measurement error included in the error vector.

### 2.3. Evaluation Procedure

The potential of the proposed methodology is demonstrated in a numerical and experimental evaluation. For this evaluation procedure, a stiffness approximation is performed on a glass fiber-reinforced plate that is manufactured by vacuum infusion processing. First, a numerical evaluation is performed, in which conclusions are drawn on (i) the convergence of the ABD-approximation as function of the number of reference laminates (*R*) and (ii) the robustness of the algorithm as function of the number of unknown ABD-components (*S*). The findings of this numerical evaluation are used in the experimental evaluation in which the stiffness of the manufactured panel is assessed by measuring the UGW velocities. Afterwards, the test panel will be cut into test coupons and subjected to bending and tensile tests to obtain the stiffness properties according to mechanical testing.

#### 2.3.1. Plate Specifications

**Figure 2:**
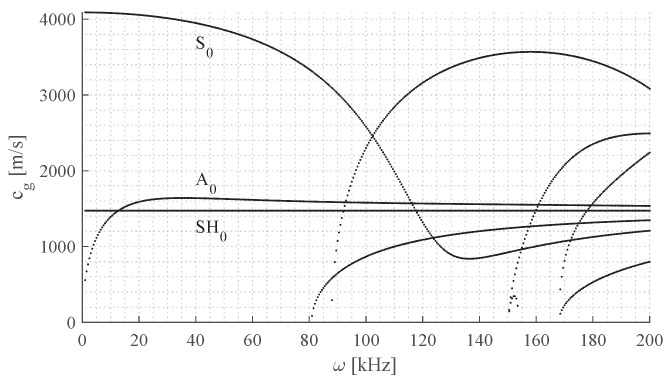
Group speed dispersion curves for the produced glass fiber-reinforced laminate with wave propagation in the 0°-direction. The $S_{0}$, $A_{0}$, and $SH_{0}$ wave modes are labeled, higher order waves modes emerge above 80 kHz.

The plate in this investigation is a cross-ply laminate consisting of transversely isotropic plies made of glass fibers and vinyl ester resin with the specifications given in Table 1. The general properties of the panel are given in Table 2. The expected ply properties are provided by manufacturing and are given in Table 3. Based on these expected plate properties, the dispersion curves are derived using SAFEM and presented in Figure 2.

#### 2.3.2. System Configuration

Equation (3) is established for waves propagating at three different frequencies and along five different directions, resulting in a total of 30 equations included in the system of equations of Equation (13) (2W=30). Given the symmetric and balanced cross-ply layup, it can be inferred that there is no coupling stiffness ($B_{ij}=0$), no stretching–shearing coupling ($A_{16}=A_{26}=0$), and no bending–twisting coupling ($D_{16}=D_{26}=0$). As a result, coefficient matrix $\left[C\right]$ in Equation (13) reduces in dimensions to $\left[2W\times S\right]=\left[30\times 8\right]$.

##### Reference Laminates

For this evaluation procedure, the stacking sequence, ply thickness ($t_{ply}$), and density (ρ) of the test panel are assumed to be known and the panel is defect-free. Furthermore, it is assumed that the actual properties of the plies ($E_{1}$, $E_{2}=E_{3}$, $G_{12}=G_{13}$, $G_{23}$, $\nu_{12}=\nu_{13}$, and $\nu_{23}$) are unknown but located within a range of ±20% with respect to a set of expected ply properties (the so-called baseline laminate). The unknown ply stiffness properties are arbitrarily generated within the range of expected ply properties. This arbitrary process is for $E_{1}$ described as

(18) $$\begin{array}{c} E_{1,p_{r}}=E_{1,BL}+\Delta E_{1}\;\;\;\mathrm{where},\;\;\Delta E_{1}=\alpha_{E_{1}}\cdot E_{1,BL} \end{array}$$

Here, $E_{1,p_{r}}$ is the randomly generated value of $E_{1}$ for the reference laminate $p_{r}$, $E_{1,BL}$ is the value of $E_{1}$ of the baseline laminate, and $\alpha_{E_{1}}$ is a randomly generated value between −20% and +20%. The same approach is used for all unknown ply properties. The ply properties of each reference laminate $p_{r}$ are randomly generated, independently of the other reference laminates. In this manner a set of 3100 reference laminates is generated. It is expected that this amount sufficiently covers the range of stiffness possibilities.

For this evaluation procedure, two sets of reference laminates are generated, each using a different baseline laminate. The first set of reference laminates (the manufacturer’s set) uses the expected ply properties provided by the manufacturer (Table 3). The second set (the mechanical testing set) uses the ply properties according to the mechanical tensile and four-point bending tests, which will be discussed in Section 4.2

#### 2.3.3. Robustness of the Algorithm

The robustness of the algorithm with respect to imperfect input data is investigated in the numerical evaluation. Three system configurations are considered, as defined in Table 4. Configuration 1 includes all the unknown stiffness components of the panel under investigation. In configurations 2 and 3, a subset of these components has been selected to shed light on the possibilities of reducing the system size based on the expected relationship between stiffness components and wave modes.

## 3. Experiments

The procedure for measuring the ultrasonic guided waves on the test panel and the performance of mechanical tests afterwards are described in Section 3.1 and Section 3.2, respectively.

### 3.1. Ultrasonic Guided Wave Testing

**Figure 3:**
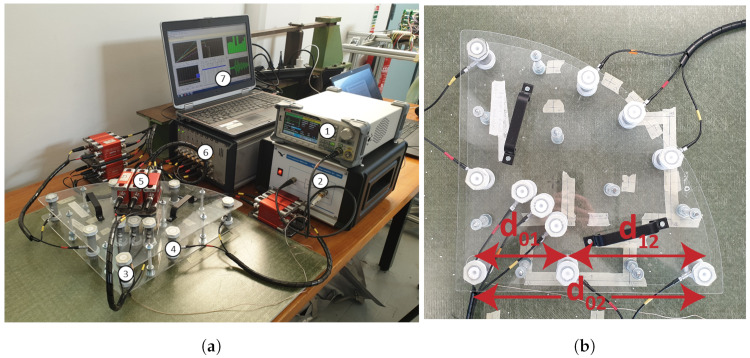
(**a**) Overview of the experimental setup including the (1) RS PRO RSDG1032X wave generator, (2) Falco Systems WMA-300 high-voltage amplifier, (3) Vallen Systeme VS600-Z1 actuator, (4) ACS Group S2803 dry-point contact transducers, (5) Vallen Systeme AEPH5 pre-amplifiers, (6) Vallen Systeme AMSY-6 data acquisition system chassis type MB6, and (7) Vallen Systeme AE-Suite software version R2023.1218.2. (**b**) Topview of the measurement device.

An overview of the experimental setup for measurement of the ultrasonic guided waves is provided in Figure 3a. A close-up of the measurement device is given in Figure 3b. The waveform generator creates an input wave signal that is amplified and emitted through a piezoelectric transducer, indicated as the actuator in Figure 3a. In total, three different wave signals are used. These signals are narrow-banded Hann-windowed sinusoidal pulses with a center frequency of 70, 80, and 90 kHz. The signals are recorded in five directions (0°, 30°, 45°, 60°, and 90° with respect to the reference axis of the laminate) by two dry point contact transducers. Each measurement set consists of a total of 30 input signals that are emitted and recorded one after another and then averaged. This procedure helps to improve the signal-to-noise ratio and mitigates the presence of background noise components in the signal. Moreover, all experiments are conducted at room temperature (20 °C), aligning with the conditions under which the reference velocities are calculated. Temperature variation was below 1 °C during the measurement period, making the effect on wave speed insignificant [58].

**Figure 4:**
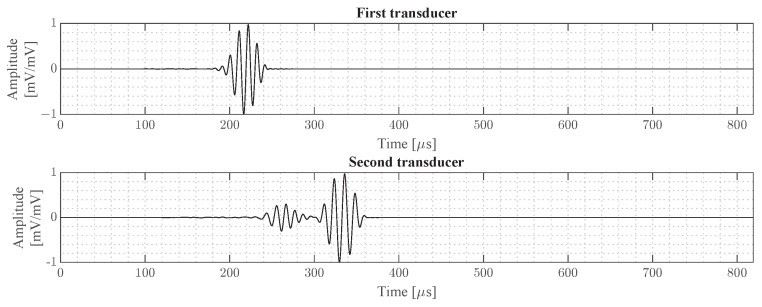
Example of an averaged wave signal recorded by the two transducers. The signal corresponds to a 80 kHz wave propagating in the 0°-direction.

An example of an averaged wave signal recorded by the two transducers is given in Figure 4. This signal corresponds to an 80 kHz wave propagating in the 0°-direction. In this figure, the dispersion effect of the $S_{0}$ wave, propagating faster than the $A_{0}$ wave, is clearly visible. Based on the arrival time of the wave at both transducers, the group wave speed of the $A_{0}$ and $S_{0}$ wave modes can be calculated using Equations (19) and (20), respectively.

(19) $$\begin{array}{c} c_{g,A_{0}}=\frac{d_{12}}{t_{A_{0}2}-t_{A_{0}1}} \end{array}$$

(20) $$\begin{array}{c} c_{g,S_{0}}=\frac{1}{\frac{1}{c_{g,A_{0}}}-\frac{t_{S_{0}2}-t_{A_{0}2}}{d_{02}}} \end{array}$$

Here, $t_{A_{0}1}$ and $t_{A_{0}2}$ represent the arrival time of the $A_{0}$ wave mode at the first and second transducer, respectively; $t_{S_{0}2}$ represent the arrival time of the $S_{0}$ wave mode at the second transducer. Lastly, $d_{12}$ and $d_{02}$ denote the distance between the first and second transducer and between the actuator and the second transducer; respectively, see Figure 3b.

### 3.2. Mechanical Testing

To assess the obtained stiffness properties using UGW testing, the test panel is subjected to mechanical testing. Test coupons are cut from the panel and subjected to both tensile and four-point bending tests to determine the stiffness components $A_{11}$, $A_{22}$, $D_{11}$, and $D_{22}$. The tests were performed in accordance with ASTM D3039/D3039M [59] and ASTM D6272 [60] with a minor deviation in the coupon dimensions driven by the limitations of the available test facilities. The coupon dimensions for the tensile and four-point bending tests were 150×50 mm and 130×25 mm, respectively. Nevertheless, these dimensions are considered reasonable and not expected to have influenced the outcomes.

**Figure 5:**
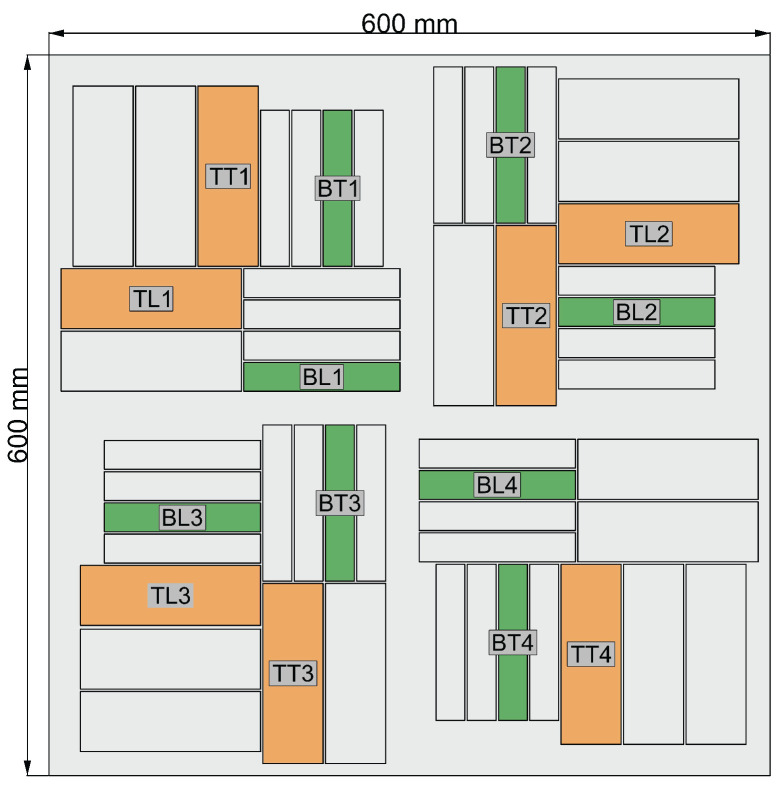
Cutting plan mechanical test coupons.

**Figure 6:**
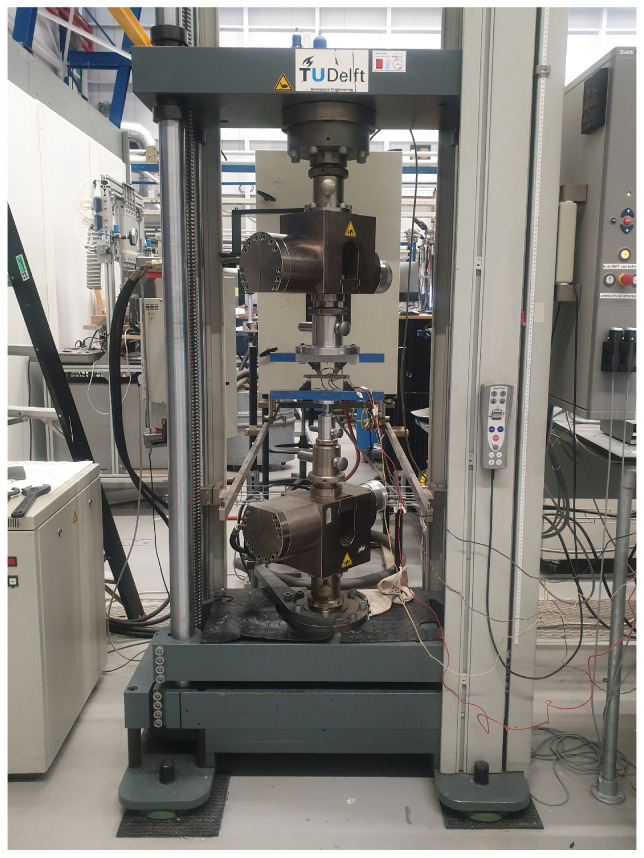
The Zwick 250 kN test bench.

**Figure 7:**
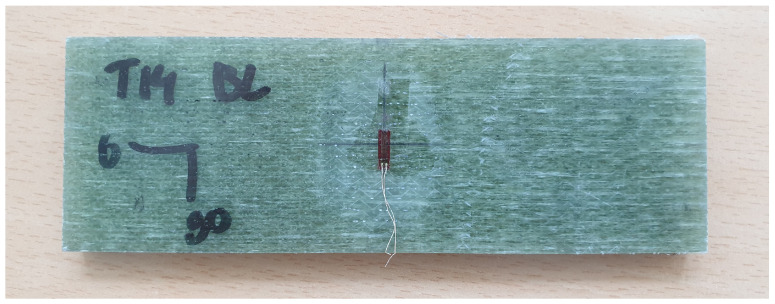
Tensile test coupon.

**Figure 8:**
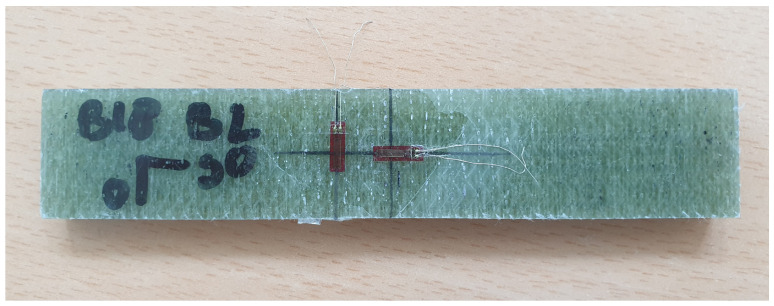
Four-point bending test coupon.

**Figure 9:**
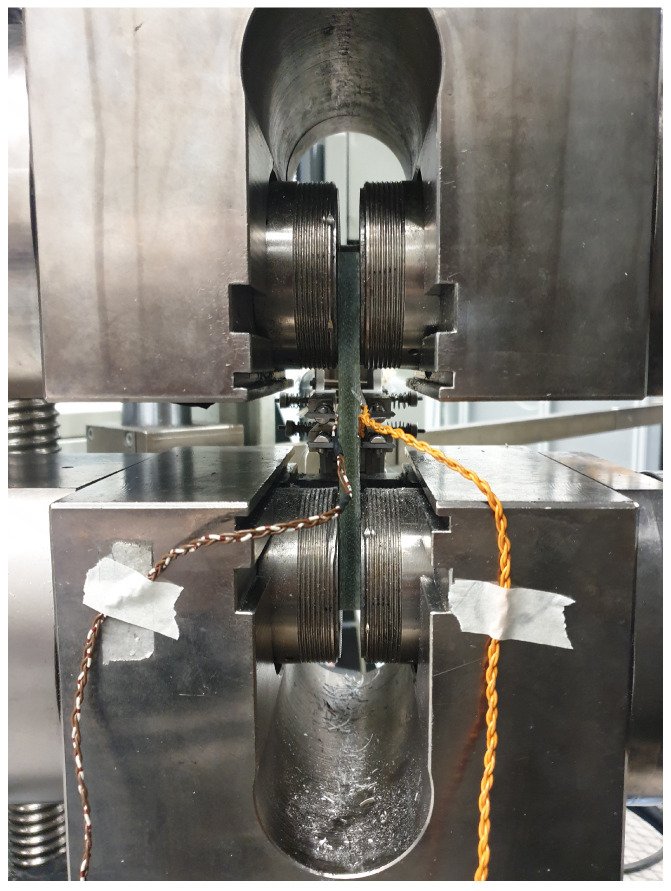
Tensile test coupon clamped in the test bench.

**Figure 10:**
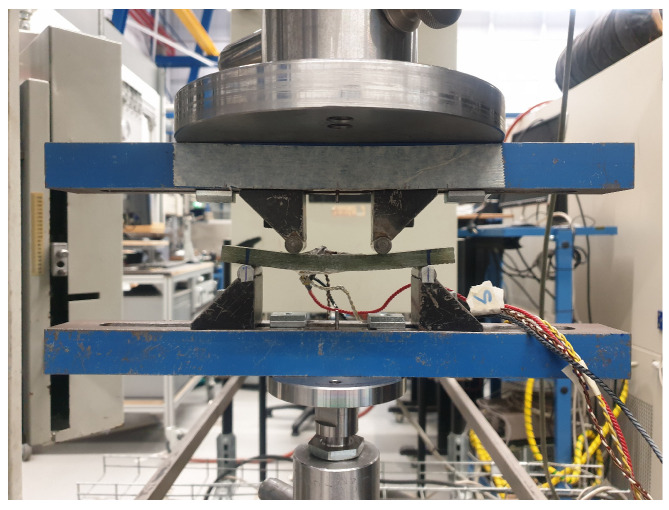
Four-point bending test coupon positioned in the test setup.

The cutting plan of the panel is depicted in Figure 5. Here, orange coupons represent those used for tensile testing and green coupons are utilized for four-point bending testing. The mechanical tests are carried out on a test bench (Figure 6) that has a maximum tensile capacity of 250 kN. In Figure 7 and Figure 8, pictures of a tensile and bending test coupon are provided, respectively. During the tensile tests, axial strain is measured using an extensometer, while transverse strain is measured using strain gauges. During the four-point bending tests, only strain gauges are used to measure longitudinal and transverse strain. Unidirectional strain gauges are employed, requiring the transverse strain gauges to be placed slightly off-center, as depicted in Figure 8. Nevertheless, it is considered that the measured strain at these positions is indicative of the strain at the center of the coupon. Figure 9 and Figure 10 illustrate a tensile and bending coupon, respectively, during the measurement.

## 4. Results and Discussion

### 4.1. Numerical Evaluation

In the numerical evaluation, the convergence of the stiffness approximation as function of the number of reference laminates (*R*) and the robustness of the algorithm as function of the number of unknown ABD-components (*S*) are investigated. The manufacturer’s set of reference laminates is used for the numerical evaluation.

#### 4.1.1. Convergence Study

For the convergence study, system configuration 1 of the robustness study (Table 4) is used. Each reference laminate $p_{r}$ included in the manufacturer’s set is used as test case for the convergence study. Velocity-squared vector $\left\{c_{g}^{2}\right\}$, which is calculated using SAFEM, is input for the methodology; see Equation (4). For each test case, the size of *R* is increased from 2 up to 3100 reference laminates to conclude what set size is sufficient to obtain a converged ABD approximation. For each value of *R*, the reference laminates $p_{r}$ are arbitrarily selected from the manufacturer’s set. The approximated ABD-components ($\Psi_{r}$) are compared to the results according to the CLT ($\Psi_{{\mathrm{ref}}_{n}}$). Eventually, the mean absolute percentage error (MAPE) of the total of test cases (TC) is calculated as follows:

(21) $$\begin{array}{c} \mathrm{MAPE}=\frac{\sum_{r=2}^{TC}\left|\frac{\Psi_{r}}{\Psi_{{\mathrm{ref}}_{r}}}\right|\cdot 100\%}{TC}\;\;\mathrm{where},\;\;r\in \left[2,3100\right] \end{array}$$

**Figure 11:**
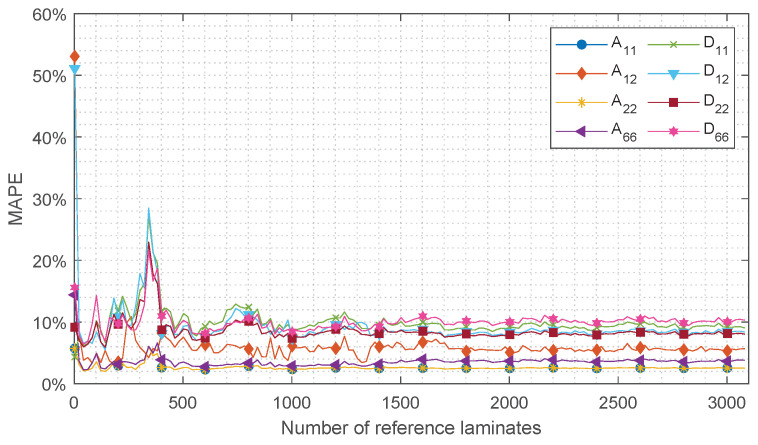
Convergence study with respect to the size (*R*) of the manufacturer’s set of reference laminates. The MAPE of 3100 test cases compared to CLT is calculated using Equation (21).

**Figure 12:**
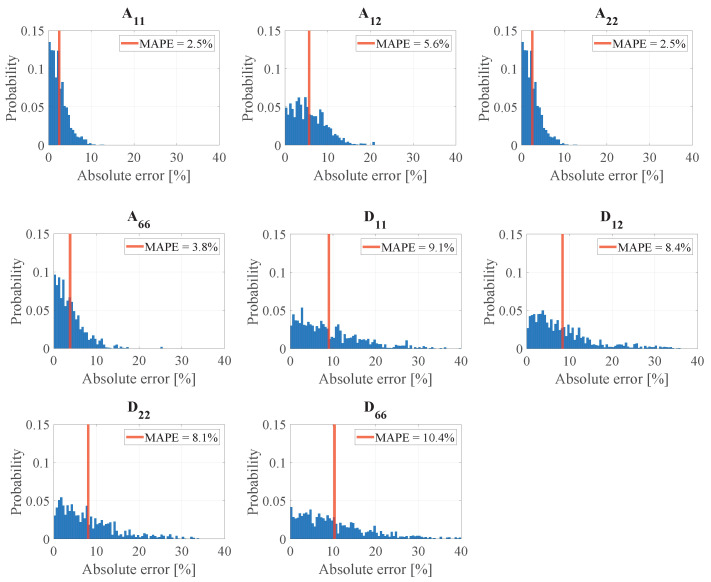
The distribution of the absolute error for each ABD-components for the 3100 test cases as well as the MAPE value.

The results of the convergence study are shown in Figure 11. It can be observed that the approximation of all ABD-components is converged around 2000 reference laminates. The approximation of components $A_{11}$ and $A_{22}$ shows the fastest convergence around 1500 reference laminates. In the figure, significant peaks are observed in the range of *R* lower than 1000 reference laminates. These peaks are mainly the result of the arbitrary selection procedures of the reference laminates $p_{r}$ included in *R* and indicate insufficient coverage of the range of stiffness possibilities. Repeating this convergence study with again an arbitrary $p_{r}$ selection will lead to a shift in the peak locations with respect to *R*. The generated set of 3100 reference laminates is considered sufficient to obtain converged results. In Figure 12, the error distributions of the test cases are shown as well as the MAPE value for each ABD-component. These results are obtained using the complete set of 3100 reference laminates. It is shown that each ABD-component can be approximated within a MAPE of 10.4%. The pure extensional and bending stiffness properties can be approximated with an average MAPE of 3.6% and 9.1%, respectively. This difference in MAPE between the extensional and bending stiffness properties may be related to the quality of the approximation in Equation (3), leading to a larger error for the antisymmetric wave modes than for the symmetric wave modes.

#### 4.1.2. Robustness of the Algorithm

The sensitivity of the algorithm on measurement errors in the UGW input data is evaluated by analyzing the MAPE (Equation (21)) of the approximated $A_{11}$, $A_{22}$, $D_{11}$, and $D_{22}$ stiffness components for increasing $E_{max}$. For this study the complete set of 3100 reference laminates is used and the range of $E_{max}$ is set from 0% to 10%.

**Figure 13:**
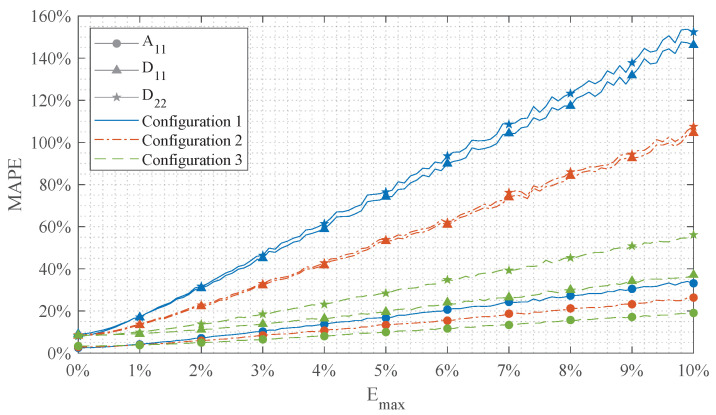
Sensitivity study on the robustness of the algorithm on measurement errors in the input data. The MAPE of 3100 test cases compared to CLT is calculated using Equation (21).

**Figure 14:**
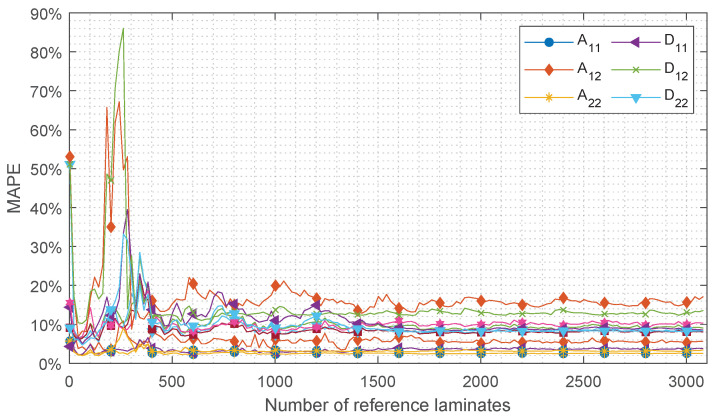
Convergence study with respect to the size of the manufacturer’s set of reference laminates of system configuration 3. The MAPE of 3100 test cases compared to CLT is calculated using Equation (21).

The results are presented in Figure 13. The results indicate that system configuration 3 (Table 4) is least sensitive to measurement errors and can offer the best robustness in practical environments. Configuration 3, like configuration 1, provides a converged stiffness approximation for a set size of 3100 reference laminates, as illustrated in Figure 14. Therefore, this configuration is employed in the experiments, implying that $\left[C\right]$ and $\bar{\Psi }$ of Equation (13) are of dimensions $\left[2W\times S\right]=\left[30\times 6\right]$ and $\left[1\times S\right]=\left[1\times 6\right]$, respectively.

### 4.2. Experimental Evaluation

#### 4.2.1. Stiffness Assessment by Mechanical Testing

The results of the tensile tests and four-point bending tests are presented in Table 5 and Table 6, respectively.

**Figure 15:**
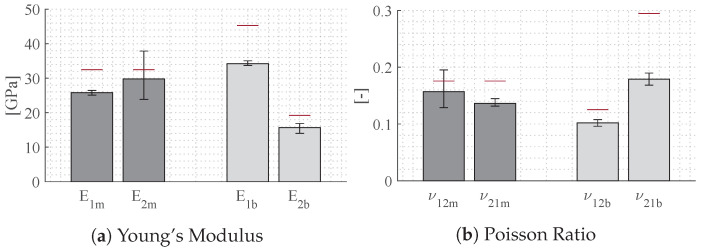
Results of mechanical testing. The expected laminate stiffness properties of the plate of interest according to manufacturing are indicated by the red lines.

Figure 15 displays the results of the mechanical tests, along with the expected laminate stiffness properties of the plate of interest according to manufacturing (Table 3) represented as red lines. These expected properties according to manufacturing are calculated using the following equations [61]:

(22) $$\begin{array}{c} E_{1m}=\frac{1}{ha_{11}}\;\;\;E_{2m}=\frac{1}{ha_{22}}\;\;\;\nu_{12m}=-\frac{a_{12}}{a_{22}}\;\;\;\nu_{21m}=-\frac{a_{12}}{a_{11}} \end{array}$$

(23) $$\begin{array}{c} E_{1b}=\frac{12}{h^{3}d_{11}}\;\;\;E_{2b}=\frac{12}{h^{3}d_{22}}\;\;\;\nu_{12b}=-\frac{d_{12}}{d_{22}}\;\;\;\nu_{21b}=-\frac{d_{12}}{d_{11}} \end{array}$$

Here, *h* denotes the thickness of the plate, and $a_{ij}$ and $d_{ij}$ denote the elements of the inverse ABD-matrix which is calculated using CLT and the properties in Table 3.

The tensile properties in the 0°-direction ($E_{1m}$) and the bending properties in both the 0°- and 90°-directions ($E_{1b}$ and $E_{2b}$, respectively) show a reasonable variation. However, there is a larger variation in the tensile properties of the 90°-coupons ($E_{2m}$). Furthermore, the extensional stiffness $E_{1m}$ is 13% lower than $E_{2m}$, indicating that the actual laminate does not behave as a balanced laminate as initially assumed. Lastly, the higher stiffness properties of the laminate of interest (red lines) suggest that the overall stiffness of the sample plate is lower than expected according to the manufacturer’s data.

##### Mechanical Testing Set of Reference Laminates

Using the findings from the mechanical tests, the mechanical testing set of reference laminates is constructed. The assumption for this set is that the lower overall stiffness properties are caused by lower $E_{1}$ and $E_{2}$ values for all plies. Additionally, it is assumed that the stiffness imbalance is caused by a difference in $E_{1}$ between the 0°- and 90°-plies, while all other known and unknown properties remain the same as those in the manufacturer’s set (Table 3). The resulting ply stiffness properties for the laminate of interest, used as baseline laminate, in the mechanical testing set of reference laminates are provided in Table 7.

#### 4.2.2. Stiffness Assessment by Ultrasonic Guided Waves Testing

**Figure 16:**
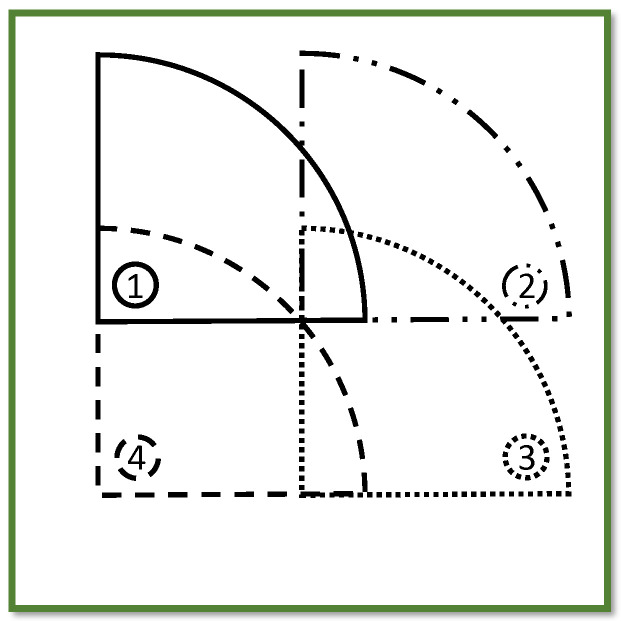
The four assessed locations on the plate labeled as 1-4 and distinguished by different line patterns.

The stiffness assessment using UGW is performed at four distinct locations on the panel, as depicted in Figure 16. These locations are selected with the consideration to minimize interference of the emitted wave signals with reflections from the boundaries of the structure. At each location, the average group wave speed is determined of 30 emitted wave signals.

**Figure 17:**
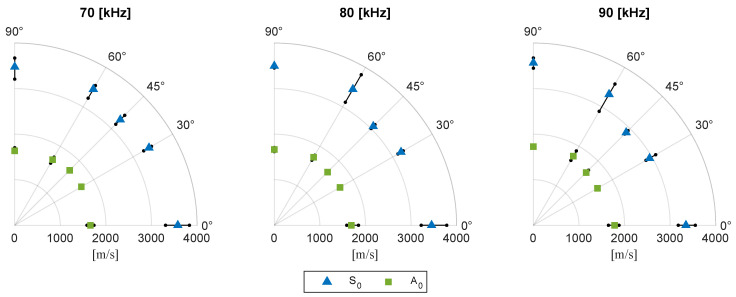
The mean $A_{0}$ and $S_{0}$ group wave speeds along with their range, measured at the four locations on the panel.

The resulting mean group wave speeds and the range over the four locations are presented in Figure 17. The figure indicates that there are minimal variations in wave speed of the $A_{0}$ mode across the panel. The wave speed of $S_{0}$ exhibits greater variations across the panel.

##### Results Using the Manufacturer’s Set of Reference Laminates

**Figure 18:**
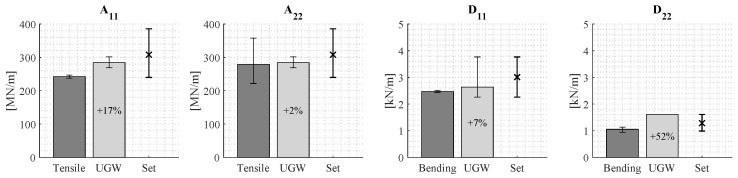
The approximated stiffness properties using the set of reference laminates based on the manufacturer’s data (UGW) as well as the stiffness properties according to mechanical testing (Tensile & Bending). Also, the range of stiffness properties included in the set of reference laminates (Set) is shown.

Figure 18 displays the stiffness properties approximated by implementing the algorithm using the manufacturer’s set of reference laminates (labeled as UGW). The figure also shows the results of mechanical testing (labeled as Tensile and Bending) and the range of stiffness properties included in the set of reference laminates (labeled as Set). The extensional stiffness $A_{22}$ as well as the bending stiffness $D_{11}$ show reasonable agreement with the mechanical tests, with an average deviation of +2% and +7%, respectively. However, extensional stiffness $A_{11}$ and bending stiffness $D_{22}$ have a larger deviation from mechanical testing, being +17% and +52%, respectively. Furthermore, the range of $D_{11}$ is relatively large, indicating the algorithm approximates the stiffness properties with considerable variations across the panel. Lastly, it can be observed that the laminate stiffness properties according to mechanical testing only marginally fall within the range of stiffness properties included in the set of reference laminates.

##### Results Using the Mechanical Testing Set of Reference Laminates

**Figure 19:**
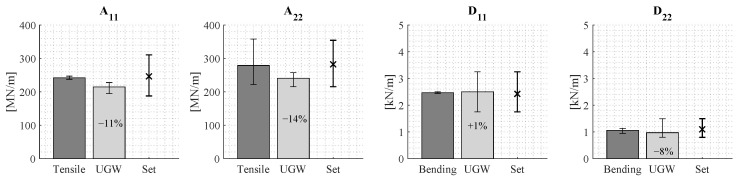
The approximated stiffness using the set of reference laminates based on the mechanical testing data (UGW) as well as the stiffness properties according to mechanical testing (Tensile and Bending). Also, the range of stiffness properties included in the set of reference laminates (Set) is shown.

Figure 19 presents the stiffness approximation using the mechanical testing set of reference laminates. The figure demonstrates that this set of reference laminates more accurately covers the plate stiffness properties according to mechanical testing. Moreover, the approximation of $D_{22}$ shows a significant improvement. Nonetheless, notable differences between the approximated stiffness properties using UGW testing and those obtained from mechanical testing still exist. Furthermore, the results obtained using the algorithm show large variations in the stiffness approximation for $D_{11}$ and $D_{22}$.

These differences in stiffness assessment between the algorithm and mechanical testing, as well as the wide range of approximated stiffness properties by the algorithm, can be partly attributed to the moderate quality of the manufactured test panel. Orientation of plies may be subject to inaccuracy and additionally the plies may not be perfectly transversely isotropic. Mechanical testing revealed that stiffness variations were mainly observed in $E_{2m}$ across the panel. Furthermore, the area over which a single measurement is performed (Figure 16) is relatively large compared to the size of the panel and the coupons used for mechanical testing (Figure 5). As a result, the stiffness variations across the measured area are averaged by the measurements, and the algorithm provides an average stiffness approximation of this measured area. Therefore, an ideal comparison of the stiffness properties of a single mechanical testing coupon to those of the algorithm is not possible. To achieve this, the minimal required area for UGW testing should be reduced; this might pose, however, additional challenges in separating the two fundamental wave modes, as well as performing measurements along multiple directions.

### 4.3. Limitations and Practical Remarks

The methodology presented in this study estimates the average stiffness properties over the measurement area, which is determined by the position of the source and receivers. Consequently, if any damaged area of interest is not covered by the wave propagation path, no change in the stiffness properties will be detected either. In addition, defects that are present in the measurement area will be captured by their effect on the average stiffness properties. It is believed that this will not hamper the intended applications of the methodology for rapid inspection or scanning of designated areas of large-scale composite structures.

When applying the methodology in practice, the influence of environmental conditions should be taken into account. At first, the effect of temperature on wave propagation velocities should be considered. In the experimental evaluation and as mentioned earlier, all experiments were conducted at room temperature (20 °C). In case measurements are performed at notably different temperatures, application of temperature compensation algorithms for the extracted wave velocities may be necessary. Examples of such algorithms can be found in literature [62,63,64,65,66]. Secondly, the presence of background noise interfering with the input signal may influence the measurement accuracy. To mitigate the presence of background noise components, e.g., due to environmental and electromagnetic interference, a total of 30 signals were averaged in the experimental evaluation. When implementing the technology in circumstances with significant background noise, such as machinery, electrical systems, waves and wind, the number of averaged wave signals may need to be increased.

## 5. Conclusions

In this research, a new methodology for assessing the structural stiffness of FRC materials is proposed. The methodology uses an inversion algorithm that couples UGW speed to structural stiffness. The performance of the methodology is demonstrated in a numerical and experimental evaluation. For this evaluation, a glass fiber plate consisting of a symmetric and balanced cross-ply layup is manufactured. The following conclusions are drawn from the numerical evaluation:

- The stiffness approximation provides converged results when the size of the set of reference laminates is sufficiently large. A set of 2000 reference laminates is concluded sufficient for the stiffness assessment of a balanced and symmetric laminate for which the actual properties of the plies are known within a range of ±20%. The numerical evaluation showed that each ABD-component can potentially be approximated within a MAPE of 10.4% compared to its actual value for 3100 test cases. The extensional stiffness can be approximated with an average MAPE of 3.6%. The bending stiffness properties have an average MAPE of 9.1%.
- To apply the technology in typical in situ environments, it is found that a system configuration which includes all ten ABD-components is sensitive to measurement errors in the input data. To deal with this, it is concluded that a system configuration excluding the shear stiffness components $A_{66}$ and $D_{66}$ provides better system robustness against measurement errors.

The findings of the numerical evaluation are implemented in the experimental evaluation. Measurements are performed on the test panel and compared to the stiffness approximation according to mechanical testing. The results of mechanical testing revealed that the laminate was less stiff and did not exhibit the anticipated behavior of a balanced laminate, as initially assumed. Moreover, variations in $E_{2m}$ across the panel are observed. It is concluded that the stiffness assessment using the algorithm is not in desirable agreement with mechanical testing and variations across the plate are observed. These differences are partly attributed to the moderate quality of the manufactured test panel as well as the relatively large dimensions of the measurement device compared to the test coupons. In future research, the results can be improved by optimization of the measurement device and improvement of the test panel’s production quality. 

## Figures and Tables

**Table 1:** Material components used.

Component	Name	$V_{f}$/$V_{m}$
Fiber	Seartex U-E-640 g/$m^{2}$	48%
Resin	Atlac E-Nova MA 6215	52%
Hardener	Curox CM-75	-

**Table 2:** General plate properties.

Width	Length	$\rho_{\mathrm{resin}}$	$\rho_{\mathrm{fiber}}$	$\rho_{\mathrm{overall}}$	Fiber Type	$t_{\mathrm{total}}$
[mm]	[mm]	[kg/$m^{2}$]	[kg/$m^{2}$]	[kg/$m^{2}$]	[-]	[mm]
600	600	1200	2600	1872	UD 600	9.30

**Table 3:** The expected ply properties of the test panel according to manufacturing.

$E_{1}$	$E_{2},E_{3}$	$G_{12},G_{13}$	$G_{23}$	$\nu_{12},\nu_{13}$	$\nu_{23}$	Layup	$t_{\mathrm{ply}}$	ρ
[GPa]	[GPa]	[GPa]	[GPa]	[-]	[-]	[-]	[mm]	[kg/$m^{3}$]
46.2	13.1	4.1	5.1	0.29	0.28	${\left[0_{5}/90_{5}\right]}_{S}$	0.465	1872

**Table 4:** The system configurations considered for the numerical sensitivity study.

Configuration	{Ψ}
1	$\left\{\begin{array}{ccccccccc} A_{11} & A_{12} & A_{22} & A_{66} & | & D_{11} & D_{12} & D_{22} & D_{66} \end{array}\right\}$
2	$\left\{\begin{array}{ccccccc} A_{11} & A_{22} & A_{66} & | & D_{11} & D_{22} & D_{66} \end{array}\right\}$
3	$\left\{\begin{array}{ccccccc} A_{11} & A_{12} & A_{22} & | & D_{11} & D_{12} & D_{22} \end{array}\right\}$

**Table 5:** Results of tensile tests.

		Coupons	Mean	std
$E_{1m}$	[GPa]	3	25.81	0.69
$E_{2m}$	[GPa]	4	29.78	6.47
$\nu_{12m}$	[-]	3	0.14	0.01
$\nu_{21m}$	[-]	4	0.16	0.03

**Table 6:** Results of four-point bending tests.

		Coupons	Mean	std
$E_{1b}$	[GPa]	3	34.21	0.58
$E_{2b}$	[GPa]	4	15.68	1.31
$\nu_{12b}$	[-]	3	0.18	0.01
$\nu_{21b}$	[-]	4	0.10	0.01

**Table 7:** Ply stiffness properties for the baseline laminate in the mechanical testing set of reference laminates.

$E_{1}$	$E_{2}$,$E_{3}$	$G_{12}$,$G_{13}$	$G_{23}$	$\nu_{12}$, $\nu_{13}$	$\nu_{23}$	Layup	$t_{\mathrm{ply}}$	ρ
[GPa]	[GPa]	[GPa]	[GPa]	[-]	[-]	[-]	[mm]	[kg/$m^{3}$]
29.6 (0°)								
39.6 (90°)	10.5	4.1	5.1	0.29	0.28	${\left[0_{5}/90_{5}\right]}_{S}$	0.465	1872

## Data Availability

The data presented in this study are available upon request from the corresponding author.
